# Retinal Thickness Changes Over Time in a Murine AD Model APP^**NL-F/NL-F**^

**DOI:** 10.3389/fnagi.2020.625642

**Published:** 2021-01-15

**Authors:** Elena Salobrar-García, Inés López-Cuenca, Lídia Sánchez-Puebla, Rosa de Hoz, José A. Fernández-Albarral, Ana I. Ramírez, Isabel Bravo-Ferrer, Violeta Medina, María A. Moro, Takaomi C. Saido, Takashi Saito, Juan J. Salazar, José M. Ramírez

**Affiliations:** ^1^Ramon Castroviejo Ophthalmological Research Institute, Complutense University of Madrid, Madrid, Spain; ^2^Department of Immunology, Ophthalmology and Ear, Nose, and Throat, Faculty of Optics and Optometry, Complutense University of Madrid, Madrid, Spain; ^3^Department of Pharmacology and Toxicology, Faculty of Medicine, Complutense University of Madrid, Madrid, Spain; ^4^Edinburgh Medical School, UK Dementia Research Institute, University of Edinburgh, Edinburgh, United Kingdom; ^5^Centro Nacional de Investigaciones Cardiovasculares (CNIC), Madrid, Spain; ^6^Laboratory for Proteolytic Neuroscience, Brain Science Institute, RIKEN, Wako, Japan; ^7^Department of Neurocognitive Science, Institute of Brain Science, Nagoya City University Graduate School of Medical Sciences, Nagoya, Japan; ^8^Department of Immunology, Ophthalmology and ENT, School of Medicine, Complutense University of Madrid, Madrid, Spain

**Keywords:** Alzheimer, retina, OCT, mouse model of AD, APPNL-F/NL-F

## Abstract

**Background:** Alzheimer's disease (AD) may present retinal changes before brain pathology, suggesting the retina as an accessible biomarker of AD. The present work is a diachronic study using spectral domain optical coherence tomography (SD-OCT) to determine the total retinal thickness and retinal nerve fiber layer (RNFL) thickness in an APP^NL−F/NL−F^ mouse model of AD at 6, 9, 12, 15, 17, and 20 months old compared to wild type (WT) animals.

**Methods:** Total retinal thickness and RNFL thickness were determined. The mean total retinal thickness was analyzed following the Early Treatment Diabetic Retinopathy Study sectors. RNFL was measured in six sectors of axonal ring scans around the optic nerve.

**Results:** In the APP^NL−F/NL−F^ group compared to WT animals, the total retinal thickness changes observed were the following: (i) At 6-months-old, a significant thinning in the outer temporal sector was observed; (ii) at 15-months-old a significant thinning in the inner temporal and in the inner and outer inferior retinal sectors was noticed; (iii) at 17-months-old, a significant thickening in the inferior and nasal sectors was found in both inner and outer rings; and (iv) at 20-months-old, a significant thinning in the inner ring of nasal, temporal, and inferior retina and in the outer ring of superior and temporal retina was seen. In RNFL thickness, there was significant thinning in the global analysis and in nasal and inner-temporal sectors at 6 months old. Thinning was also found in the supero-temporal and nasal sectors and global value at 20 months old.

**Conclusions:** In the APP^NL−F/NL−F^ AD model, the retinal thickness showed thinning, possibly produced by neurodegeneration alternating with thickening caused by deposits and neuroinflammation in some areas of the retina. These changes over time are similar to those observed in the human retina and could be a biomarker for AD. The APP^NL−F/NL−F^ AD model may help us better understand the different retinal changes during the progression of AD.

## Introduction

Alzheimer's disease (AD) is a neurodegenerative brain pathology characterized by a loss of neurons and their synapses, after which an atrophy of the cerebral cortex develops (Sharma and Singh, 2016). The main features of AD are extracellular deposits of the protein amyloid-β (Aβ), the formation of plaques, and intraneuronal hyper-phosphorylated tau (pTau) in the form of neurofibrillary tangles (Ghiso et al., 2013) leading to a neuroinflammatory process (Pan et al., 2011).

One important tissue to focus on in search of neurodegenerative disease biomarkers is the eye. It is widely known that patients with AD have visual problems, such as decreased visual acuity, contrast sensitivity, color perception, and visual integration (Salobrar-García et al., 2015a, 2016, 2019a,b). Retinal changes have also been documented *in vivo* using optical coherence tomography (OCT) both in humans (Iseri et al., 2006; Garcia-Martin et al., 2014; Salobrar-García et al., 2015b, 2016, 2019a,b; Polo et al., 2017; Ko et al., 2018; Lad et al., 2018) and in different AD animal models (Chiquita et al., 2019; Georgevsky et al., 2019; Harper et al., 2020). The retinal changes observed in the OCT results of AD patients showed macular thinning when the disease is in an early stage, followed by thinning of the peripapillary area when AD progresses (Garcia-Martin et al., 2014; Salobrar-García et al., 2015b, 2019a,b; Jáñez-Escalada et al., 2019). Retinal areas with an increased thickness were found in AD patients, specifically in the macular area (Jáñez-Escalada et al., 2019; Salobrar-García et al., 2019a), revealing areas of possible gliosis prior to neurodegeneration. These changes could be correlated with those found in the retinas of AD transgenic models, where marked neurodegeneration and a loss of optic nerve axons were observed (Gupta et al., 2016; Chiquita et al., 2019; Georgevsky et al., 2019), alongside retinal thickening and increased microglial activation in the early stages of the disease (Perez et al., 2009; Yang et al., 2013; Gao et al., 2015; Salobrar-García et al., 2020). In addition to the retinal structural changes, several functional changes have been observed by means of electroretinogram (ERG) in the APP_swe/PS1_ transgenic mouse model of AD finding a significant reduction of the a and b wave amplitudes between 12 and 16 months of age (Perez et al., 2009). Other authors observed in this model, already at 3 months a significant reduction of the b-wave coinciding with the Aβ deposits in the hippocampus (Georgevsky et al., 2019). In addition, findings, such as a slightly shortened ERG latency in dark adapted conditions and the increased frequency of oscillatory potentials in the old APP_swe/PS1_, could be related to inadequate cholinergic innervation (Leinonen et al., 2016). However, in this model, late-stage photopic ERG measurements revealed that the cone mediated retinal response was preserved in the APP_swe/PS1_ mice (Joly et al., 2017). Therefore, the retina has been postulated as an accessible biomarker of AD.

Most cases of AD in humans are sporadic, and only <3% are caused by genetic mutations (Selkoe, 2001). There is currently no mouse model for sporadic AD (Foidl and Humpel, 2020). In recent decades, different transgenic AD models have been generated for mice to mimic the main neuropathological hallmarks of the disease (Foidl and Humpel, 2020), but there is no transgenic mouse model that presents all AD features. Most transgenic mice were made to overexpress mutant forms of APP and/or PS1 and show the onset of Aβ age-dependent brain deposition, gliosis, synaptic dysfunction, and memory deficits (Duyckaerts et al., 2008). Transgenic mice that overexpress APP have artificial phenotypes because, in addition to the Aβ, they overproduce other APP fragments that can interfere with intrinsic biological functions. In addition, these models use artificial promoters that produce transgenic expression in cells that are not always identical to those that express endogenous APP. Another feature of APP overexpression models is the sudden death that reflects a physiological alteration (Nilsson et al., 2014; Saito et al., 2014). A second generation of AD mouse models was developed to have both less artificial phenotypes and less altered physiology (Sakakibara et al., 2018). This alternative AD mouse models have been generated via knock-in (KI) of a humanized Aβ sequences harboring familial AD mutations [Swedish (NL), Beyreuther/Iberian (F), and Arctic (G)] (Sakakibara et al., 2019). One of these second generation AD mouse models is the APP^NL−F/NL−F^ that harbors the Swedish mutation (NL) and the Iberian mutation (F). This model, unlike the models that overexpress APP, has normal levels of full-length APP, and its cleavage products produce a significantly higher level of Aβ_42_ compared to wild type (WT) mice and APP overexpression models, as well as exhibits a significantly higher Aβ_42_/Aβ_40_ ratio (Saito et al., 2014). Increased Aβ_42_ levels in this model cause pathological deposits of Aβ in the cerebral cortex and hippocampus, leading to infiltration of the microglia and astrocytes surrounding the Aβ plaques starting at 6 months old (Sasaguri et al., 2017). The APP^NL−F/NL−F^ model reproduces several key pathologies found in AD patients. It has been suggested that this model may be useful as a preclinical AD mouse model to research the pathological role of amyloidosis and amyloids related to neuroinflammation (Saito and Saido, 2018). In addition, this model presents a number of neurological disturbances in an age-dependent manner, such as synaptic disorders and memory impairment, in a Y-maze test (Saito et al., 2014).

For retinal tissue, Aβ deposits have been found in several AD models that overexpress APP (Ning et al., 2008; Shimazawa et al., 2008; Dutescu et al., 2009; Perez et al., 2009; Koronyo-Hamaoui et al., 2011; Gupta et al., 2016). These Aβ deposits were located in the retinal nerve fiber layer (RNFL), ganglion cell layer (GCL), inner plexiform layer (IPL), outer plexiform layer (OPL), and inner nuclear layer (INL) (Ning et al., 2008; Dutescu et al., 2009; Perez et al., 2009; Koronyo-Hamaoui et al., 2011).

Recently, the retina has been studied by OCT in transgenic mouse models that overexpress APP, but these studies are scarce and controversial. In an APP/PS1 model analyzing the retina from 3 to 12 months of age, a significant decrease in retinal thickness in the inner layers was found at 9 months of age and in the outer layers at 12 months (Georgevsky et al., 2019). In the 3xTg-AD animal model, neurodegeneration was found to start at 4 months-old in the innermost retinal layers. As the disease progressed, significant changes were found in every analyzed layer, with the exception of the ONL, where a thickening was observed at 12 months of age (Chiquita et al., 2019), as well as a significant thinning of the RNFL in AD mouse retinas compared to the WT controls (Song et al., 2020).

Despite the loss of neurons that occurs at ~17 months-age, for most transgenic models of AD (APP/PS1 model) (Harper et al., 2020), diachronic studies that analyze the retinal changes observed by OCT in AD transgenic models from early stages to late stages of the disease could help us better understand the retinal observations. To the best of our knowledge, there is no study that analyzes the retina using SD-OCT in the APP^NL−F/NL−F^ model. Given the aforementioned advantages of this model, the aim of this study was to analyze the changes in retinal thickness (total retinal thickness and RNFL thickness) over time (at 6, 9, 12, 15, 17, and 20 months of age) in a well-validated mouse model of AD (APP^NL−F/NL−F^).

## Materials and Methods

### Animals and Ethics

The experiments were performed on male APP^NL−F/NL−F^ mice produced by manipulating the mouse APP gene using a knock-in strategy with Swedish (KM670/671NL) and Beyreuther/Iberian (I716F) mutations, as described previously (Saito et al., 2014). The experiments were also performed on age-matched WT animals (C57BL/6J). The animals were obtained from the research group led by Dr. Takaomi C. Saito at the laboratory for Proteolytic Neuroscience, RIKEN Brain Science Institute, Saitama, Japan.

The Aβ sequence within the mouse APP gene was humanized and, while the Swedish mutation (NL) elevates the total amount of Aβ_40_ and Aβ_42_, the Beyreuther/Iberian(F) mutation increases the ratio of Aβ_42_/A_β40_ (Saito et al., 2014). The great advantage of this model is that the mouse Aβ sequence is humanized and the Swedish and Beyreuther/Iberian mutations are introduced by knock-in technology (Saito et al., 2014). In order to accelerate pathology and to remove murine endogenous Aβ, mutant mice are bred in homozygosity (Sasaguri et al., 2017) explaining why control mice are not littermates. However, this should not be a major problem considering that mutant mice have been backcrossed with genuine wild-type B6J mice for more than 10 generations.

The retinas of male APP^NL−F/NL−F^ and WT animals were evaluated *in vivo* using SD-OCT at 6, 9, 12, 15, 17, and 20 months of age.

The animals were housed in light- and temperature-controlled rooms with a 12-h light/dark cycle and *ad libitum* access to food and water in the Medical School at the University Complutense of Madrid. Light intensity within the cages ranged from 9 to 24 lux. The SD-OCT analysis was performed under general anesthesia induced with an intraperitoneal (ip) injection of a mixture of ketamine (75 mg/kg; Anesketin®, Dechra Veterinary Products SLU, Barcelona, Spain) and medetomidine (0.26 mg/kg; Medetor®, Virbac España S.A., Barcelona, Spain), which can be reversed by the antagonist atipamezole (Antisedan, 5 mg/mL; Pfizer). During the recovery from anesthesia, the mice were placed in their cages with a heat source to maintain their core body temperature.

All procedures were performed in accordance with the European Parliament, the Council Directive 2010/63/EU, and Spanish legislation (Real Decreto 53/ 2013). The procedures were approved by the Ethics Committee on Animal Welfare of the University Complutense (PROEX No. 047/16) and reported according to the Association for Research in Vision and Ophthalmology (ARVO) statement of animal use. All procedures minimized the number of animals used and their suffering.

### Experimental Groups

Two groups of mice were used for this study: an APP^NL−F/NL−F^ group (*n* = 55) and an age-matched control (WT, *n* = 41) group, as indicated in Table 1. Only the left eyes of the animals were used in our study. This control-case study was performed at 6, 9, 12, 15, 17, and 20 months of age.

**Table 1:** Number of mice used at different time points.

**Age group**	**APP^**NL-F/NL-F**^ group (*n*)**	**WT group (*n*)**
6 months	5	7
9 months	14	6
12 months	10	7
15 months	6	7
17 months	8	7
20 months	12	7

### OCT Analysis

The retinal structures were evaluated using SD-OCT Spectralis with the Heidelberg Eye Explorer software v6.13 (Heidelberg Engineering, Heidelberg, Germany) after pupil dilation (tropicamide 10 mg/ml; colircusi tropicamide, Alcon Healthcare, Barcelona, Spain).

The cornea was kept moisturized using artificial tear eye drops. To prevent a reduction in body temperature, heating pads were placed underneath the mice.

An addition, a 25 diopter mouse lens (Heidelberg, Germany) was added in front of the OCT camera, and the murine eye was covered with a polymethyl methacrylate contact lens (3.2 mm diameter, base curve 1.7; Cantor&Nissel, UK), which served to create a uniform refractive surface.

Each mouse eye was aligned with respect to the measurement beam to ensure that the optic nerve head (ONH) was at the center of the OCT analysis. To compensate for small eye movements, such as those that occur as a result of respiration, motion artifacts were minimized through real-time eye tracking in the device software.

**Figure 1:**
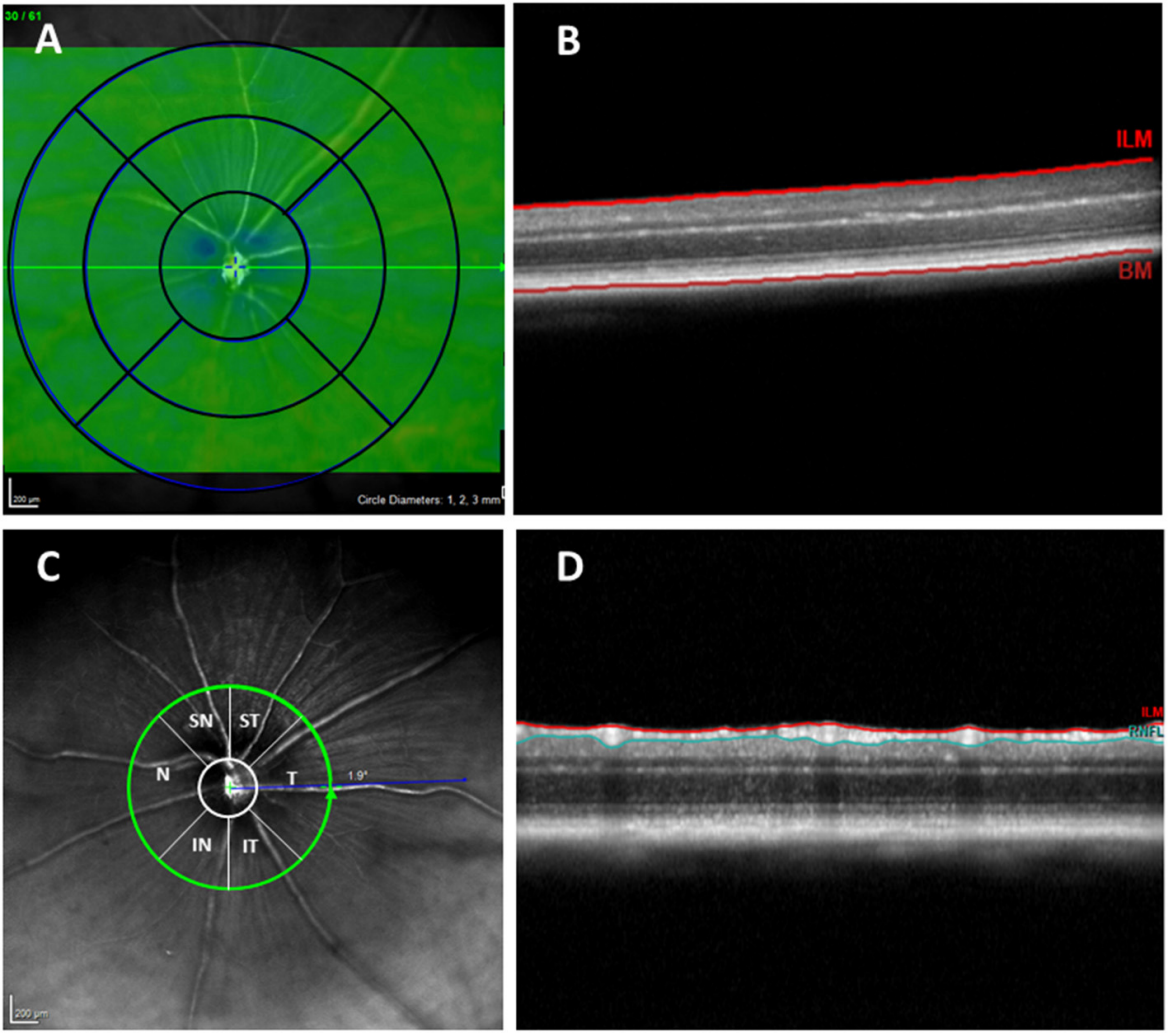
**(A)** Concentric rings with 1, 2, and 3 mm diameters. **(B)** OCT section. Retinal thickness measured between the inner limiting membrane and the retinal pigment epithelium. **(C)** RNFL sectors (RNFL, Retinal Nerve Fiber Layer; ST, Supero–Temporal; SN, Supero–Nasal; N, Nasal; IN, Infero–Nasal; IT, Infero-Temporal; T, Temporal; G, Global). **(D)** OCT section. Result of segmentation of the OCT scan. RNFL delimited between the ILM and GCL + IPL. OCT, optical coherence tomography; RNFL, Retinal nerve fiber layer; ILM, inner limiting membrane; GCL + IPL, ganglion cell layer and inner plexiform layer.

As in the Early Treatment Diabetic Retinopathy Study (ETDRS), retinal thickness data were displayed as three concentric rings 3 mm in total diameter centered in the optic nerve. These rings were distributed as follows: a central area with a diameter of 1 mm that was not considered for the measurements, an inner ring with a diameter of 2 mm, and an outer ring with a diameter of 3 mm. Both measured rings were divided into four quadrants (superior, inferior, nasal, and temporal) (Figures 1A,B). Due to the size of the mouse eye, which differs significantly from that of the human eye, a +25 diopter optical lens was used in addition to a contact lens, so the lateral distances were not entirely accurate. However, it was shown that axial measurements with OCT are accurate for the study of rodents (Dysli et al., 2015).

To analyze the RNFL, an axonal ring scan around the optic nerve head was carried out. The RNFL was measured in six sectors provided by the Heidelberg Software (Supero–Temporal, Superior, Supero–Nasal, Nasal, Infero–Nasal, Temporal, Inferior, and Temporal). The mean of all sectors is shown in the center as the global value (G) (Figures 1C,D).

The Spectralis OCT animal software allows automatic segmentation of the retinal layers and measurement of each layer's thickness through the same segmentation of the concentric circular sectors mentioned above. After collecting the images, if it was necessary to correct the automatic division of the layers, manual corrections were made by the same experienced examiner. The RNFL and total retinal thickness were then measured. The distance between the inner limiting membrane and the posterior surface of the RPE was defined as the total retinal thickness.

### Statistical Analysis

For the statistical analysis, we used the SPSS software 25.0 (SPSS Inc., Inc., Chicago, IL, USA). The differences between study groups (APP^NL−F/NL−F^ and WT) were analyzed using a non-parametric Mann–Whitney U Test. Data are reported as the mean values ± standard deviation (SD). A *P*-value < 0.05 was considered statistically significant.

## Results

In this study, we evaluated the total retinal thickness and RNFL thickness in different age groups (at 6, 9, 12, 15, 17, and 20 months) using the APP^NL−F/NL−F^ mouse model of AD (APP^NL−F/NL−F^ group) and age-matched wild-type mice (WT group). Ninety-four mice were analyzed in total−55 APP^NL−F/NL−F^ mice and 41 WT mice.

### Total Retinal Thickness

**Figure 2:**
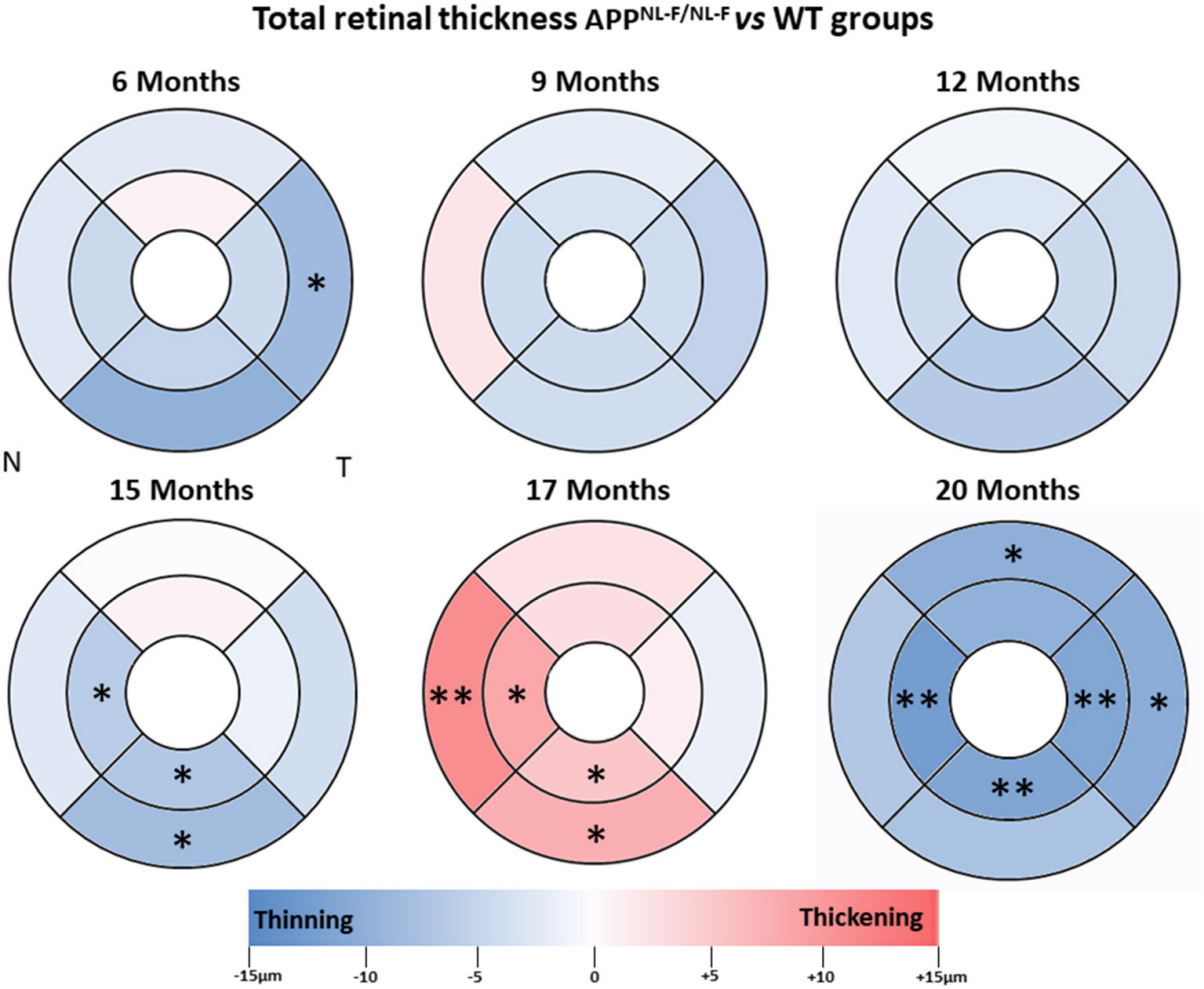
Colorimetric differences of retinal thickness in each age group between the APP^NL−F/NL−F^ and WT animal groups. OCT rings with 1, 2, and 3 mm diameters. Red: thickening; blue: thinning. APP^NL−F/NL−F^, Single App Knock-in mouse model of Alzheimer's disease; WT, Wild type; OCT, optical coherence tomography; N, nasal; T, temporal.

At 6 months of age, for the APP^NL−F/NL−F^ mice, we found that the total retinal thickness was significantly decreased in the temporal sector in the outer ring (244.40 ± 2.41) compared to the WT mice (253.00 ± 6.11; *p* < 0.05). In this same age group, compared to the WT mice, the remaining sectors showed slight thinning, except for the superior sector in the inner ring, which showed a slight thickening (without statistical significance in both cases) (Table 2, Figure 2).

**Table 2:** Total retinal thickness between groups.

			**Total retinal thickness**							
			**Inner ring**				**Outer ring**			
			**S**	**I**	**N**	**T**	**S**	**I**	**N**	**T**
6 months	APP^NL−F/NL−F^	Mean	250.60	244.20	241.40	243.00	254.20	243.80	246.20	244.40
		SD	5.73	4.82	4.28	4.53	6.38	4.44	5.63	2.41
	WT	Mean	249.43	249.43	246.00	247.43	256.57	253.57	248.86	253.00
		SD	6.02	7.30	4.28	7.25	8.28	9.11	6.18	6.11
	*P*-value		0.935	0.255	0.101	0.221	0.684	0.061	0.624	0.028^*^
9 months	APP^NL−F/NL−F^	Mean	252.36	247.29	247.43	247.57	257.71	245.36	251.21	246.14
		SD	8.42	7.24	5.26	5.56	6.78	9.43	4.51	6.68
	WT	Mean	257.17	250.33	250.50	251.83	261.67	249.00	248.17	252.33
		SD	7.31	6.12	6.92	7.68	11.38	5.37	7.36	7.12
	*P*-value		0.246	0.342	0.230	0.185	0.282	0.535	0.320	0.063
12 months	APP^NL−F/NL−F^	Mean	253.00	246.56	245.56	246.33	257.22	246.11	247.67	246.22
		SD	8.38	3.17	5.96	5.70	11.29	4.86	4.85	7.07
	WT	Mean	255.86	252.86	249.71	250.71	258.14	252.57	250.14	250.57
		SD	6.94	7.84	7.04	6.92	6.31	7.61	6.87	5.53
	*P*-value		0.366	0.089	0.202	0.123	0.560	0.100	0.243	0.110
15 months	APP^NL−F/NL−F^	Mean	253.83	248.00	244.83	249.17	257.50	247.00	248.17	247.67
		SD	4.96	7.90	2.48	8.64	9.12	7.75	4.36	9.58
	WT	Mean	252.71	254.86	251.00	250.57	257.29	255.29	250.71	251.57
		SD	7.11	5.90	6.68	8.48	9.23	6.34	6.05	6.11
	*P*-value		0.429	0.044^*^	0.031^*^	0.473	0.617	0.038^*^	0.418	0.195
17 months	APP^NL−F/NL−F^	Mean	255.38	250.25	251.63	248.25	257.75	252.88	255.00	248.13
		SD	7.48	4.37	5.73	3.37	9.10	5.30	7.48	4.52
	WT	Mean	252.29	244.71	243.00	246.71	255.00	245.00	243.71	249.71
		SD	4.68	4.57	4.73	3.20	8.50	5.45	3.30	4.68
	*P*-value		0.450	0.036^*^	0.024^*^	0.415	0.602	0.023^*^	0.004^**^	0.523
20 months	APP^NL−F/NL−F^	Mean	240.83	241.83	239.08	240.25	245.25	244.91	242.58	241.67
		SD	6.86	4.53	5.74	4.75	8.67	7.49	7.14	6.96
	WT	Mean	250.67	253.17	251.50	251.50	255.33	252.33	249.50	252.00
		SD	9.87	8.38	8.89	8.80	9.54	8.14	6.75	9.61
	*P*-value		0.054	0.007^**^	0.009^**^	0.007^**^	**0.039**	0.076	0.100	**0.031**

* in bold: p-value <0.05;

** in bold: p-value <0.01;

*Mann-Whitney U Test; WT, wild type; SD, standard deviation; S, superior; I, inferior; N, nasal; T, temporal*.

When we analyzed the 9-months-age groups, the total retinal thickness of the APP^NL−F/NL−F^ mice showed slight thinning without statistical significance in all sectors, except for the nasal sector in the outer ring, which was slightly thickened compared to the WT group (Table 2, Figure 2).

At 12 months of age, total retinal thickness in the APP^NL−F/NL−F^ group showed no statistically significant changes in comparison to the WT group. However, at 12 months old, all sectors showed slight thinning, with the inferior sectors in both the inner and outer rings being the thinnest (Table 2, Figure 2).

At 15 months of age, the total retinal thickness of the APP^NL−F/NL−F^ mice group showed a significant decrease in the inferior sector in both the inner (248.00 ± 7.90) and outer ring (247 ± 7.75) and in the inner ring of the nasal sector (244.83 ± 2.48) compared to the WT group (254.86 ± 5.90, 255.29 ± 6.34, and 251 ± 4.73, respectively *p* < 0.05 in all cases). In this age group, the remaining sectors showed slight thinning, except for the superior sector in the inner ring, which showed a slight thickening (without statistical significance in both cases), in comparison to the WT mice group (Table 2, Figure 2).

At 17 months of age, in the APP^NL−F/NL−F^ group compared to the WT group, we found a significant increase in thickness in the nasal and inferior sectors in the inner and outer rings. The nasal sectors in both the inner ring (251.63 ± 5.73 vs. 243 ± 4.73, for APP^NL−F/NL−F^ and WT groups, respectively) and the outer ring (255.00 ± 7.48 vs. 243.71 ± 3.30, for APP^NL−F/NL−F^ and WT groups, respectively) were significantly thicker (*p* < 0.05 for inner ring and *p* < 0.01 for outer ring) in the APP^NL−F/NL−F^ group compared to the WT group. Similarly, in the APP^NL−F/NL−F^ group, the inferior sectors in both the inner ring (250.25 ± 4.37 vs. 244.71 ± 4.57, for APP^NL−F/NL−F^ and WT groups, respectively) and the outer ring (252.88 ± 5.30 vs. 245.00 ± 5.45, for APP^NL−F/NL−F^ and WT groups, respectively) showed statistically significant thickening compared to the WT group (*p* < 0.05 in all cases; Table 2, Figure 2).

When we compared the APP^NL−F/NL−F^ group with the WT control group in the oldest animals (20 months), we found a significant thickness decrease in the inner and outer ring in the temporal sector (*p* < 0.01 and *p* < 0.05, respectively), in the inner ring of both inferior and nasal sectors (*p* < 0.01 in both cases) and in the outer ring of the superior sector (*p* < 0.05). In the APP^NL−F/NL−F^ mice in comparison with the WT group: the temporal sectors showed a statistically significant decrease in both the inner ring (240.25 ± 4.75 vs. 251.50 ± 8.80, in APP^NL−F/NL−F^ and WT, respectively) and in the outer ring (241.67 ± 6.96 vs. 252.00 ± 9.61, in APP^NL−F/NL−F^ and WT, respectively); both the nasal sectors and inferior sectors showed statistically significant thinning in the inner ring (239.08 ± 5.74 vs. 251.50 ± 8.89 for nasal sector and 241.83 ± 4.53 vs. 253.17 ± 8.38 for inferior sector, in APP^NL−F/NL−^F and WT, respectively) and the superior sectors showed statistically significant thinning, in the outer ring (245.25 ± 8.67 vs. 255.00 ± 9.54, in APP^NL−F/NL−F^ and WT, respectively) (Table 2, Figure 2).

### RNFL Thickness

**Figure 3:**
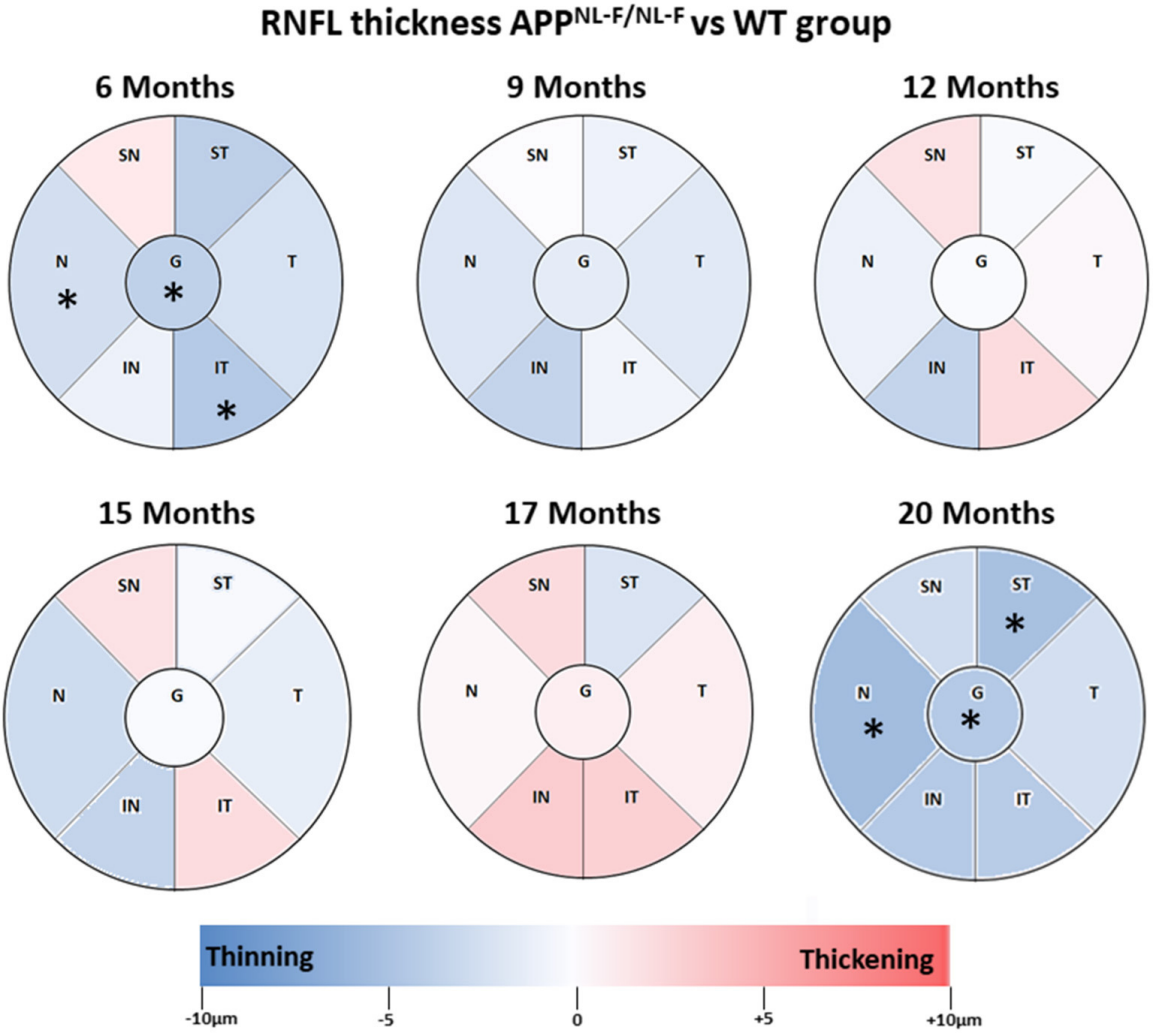
Colorimetric differences of RNFL thickness at each time point between the APP^NL−F/NL−F^ and WT groups. OCT sectors. Red: thickening; blue: thinning. APP^NL−F/NL−F^, Single App Knock-in mouse model of Alzheimer's disease; WT, Wild type; RNFL, Retinal nerve fiber layer; N, nasal; SN, Supero–Nasal; ST, Supero–Temporal; T, temporal; IT, Infero-Temporal; IN, Infero–Nasal; G, global.

Overall, in RNFL, there were no statistically significant changes over time observed in our study, except at 6 and 12 months of age. At early time point in the APP^NL−F/NL−F^ group, we found a significant thickness decrease compared to the WT group in the nasal sector (21.80 ± 1.48 vs. 24.29 ± 1.80, in APP^NL−F/NL−F^ and WT, respectively), in the infero–temporal sector (21.20 ± 1.30 *vs*. 25.57 ± 4.58, in APP^NL−F/NL−F^ and WT, respectively), and in the global value (23.00 ± 1.00 vs. 26.71 ± 4.46, in APP^NL−F/NL−F^ and WT, respectively; *p* < 0.05 in all cases) (Table 3, Figure 3).

**Table 3:** Retinal nerve fiber layer thickness between groups.

			**Retinal nerve fiber layer thickness**						
			**N**	**T**	**SN**	**ST**	**IN**	**IT**	**G**
6 months	APP^NL−F/NL−F^	Mean	21.80	23.60	25.60	25.00	24.80	21.20	23.00
		SD	1.48	0.89	4.16	2.12	1.30	1.30	1.00
	WT	Mean	24.29	25.86	24.00	28.86	25.71	25.57	26.71
		SD	1.80	2.19	3.00	4.06	4.46	4.58	4.46
	*P*-value		0.030^*^	0.062	0.741	0.087	0.868	0.014^*^	0.039^*^
9 months	APP^NL−F/NL−F^	Mean	24.29	26.36	24.64	27.00	25.57	23.86	25.29
		SD	2.73	3.46	4.09	4.71	6.28	3.63	2.61
	WT	Mean	26.17	28.00	24.50	27.83	29.00	24.50	26.67
		SD	2.14	2.37	2.95	2.64	2.37	2.43	1.75
	*P*-value		0.133	0.213	0.708	0.868	0.299	0.617	0.170
12 months	APP^NL−F/NL−F^	Mean	25.40	25.90	25.70	27.60	25.00	25.90	26.00
		SD	2.07	2.28	3.37	2.59	5.42	3.14	1.70
	WT	Mean	26.33	25.50	23.83	27.83	28.50	23.67	26.17
		SD	1.63	2.17	3.25	2.99	2.95	2.58	1.47
	*P*-value		0.352	0.659	0.414	0.869	0.190	0.272	0.868
15 months	APP^NL−F/NL−F^	Mean	24.50	25.67	25.33	26.17	28.17	25.17	25.50
		SD	3.62	1.97	3.39	4.07	4.36	2.14	2.43
	WT	Mean	27.29	26.86	26.14	29.00	29.71	25.29	27.14
		SD	3.99	3.72	3.02	5.03	2.87	5.59	2.91
	*P*-value		0.311	0.718	0.829	0.194	0.251	0.665	0.348
17 months	APP^NL−F/NL−F^	Mean	26.75	27.25	28.00	26.38	30.00	26.38	27.25
		SD	4.30	5.39	3.25	3.85	5.55	3.29	3.06
	WT	Mean	26.29	26.29	25.71	28.43	26.71	23.43	26.29
		SD	1.98	3.99	3.25	7.55	4.46	2.70	2.29
	*P*-value		0.861	0.861	0.244	0.636	0.270	0.077	0.481
20 months	APP^NL−F/NL−F^	Mean	24.83	24.58	25.25	26.33	24.83	24.17	24.67
		SD	2.33	3.73	3.47	4.08	5.56	4.84	2.06
	WT	Mean	30.33	27.00	28.00	31.50	29.17	28.33	29.00
		SD	1.86	4.52	4.86	2.35	5.42	3.44	1.79
	*P*-value		0.001^*^	0.257	0.343	0.011^*^	0.158	0.081	0.002^*^

* *in bold: p-value <0.05*,

*Mann-Whitney U Test; WT, wild type; SD, standard deviation; N, nasal; T, temporal; SN, supero-nasal; ST, supero-temporal; IN, ínfero-nasal; IT, ínfero-temporal; G, mean global thickness*.

At 9 months-old, the thickness of all sectors in the APP^NL−F/NL−F^ group slightly decreased compared to the WT group without statistical significance (Table 3, Figure 3).

By contrast, at 12 and 15 months of age, in the APP^NL−F/NL−F^ group, only four sectors showed a slight non-significant thickness decrease (in the supero–temporal, temporal, nasal, and infero–nasal sectors), and the remaining two sectors were slightly thickened without statistical significance (supero–nasal, and infero–temporal) compared to the WT group (Table 3, Figure 3).

When comparing APP^NL−F/NL−F^ and WT mice at 17 months old, it was found that the RNFL thickness of all sectors increased slightly without statistical significance, except in the supero–temporal sector, where we found a slight non-significant thickness decrease (Table 3, Figure 3).

By contrast, at 20 months of age, the highest age in the APP^NL−F/NL−F^ group, we found a significant thickness decrease in comparison to WT group in the nasal sector (24.83 ± 2.33 vs. 30.33 ± 1.86, in APP^NL−F/NL−F^ and WT, respectively), in the supero-temporal sector (26.33 ± 4.08 vs. 31.50 ± 2.35, in APP^NL−F/NL−F^ and WT, respectively), and in the global value (24.67 ± 2.06 vs. 29.00 ± 1.79, in APP^NL−F/NL−F^ and WT, respectively) (Table 3, Figure 3).

## Discussion

The present work is the first diachronic SD-OCT study of the RNFL thickness and total retinal thickness in APP^NL−F/NL−F^ mice at 6, 9, 12, 15, 17, and 20 months of age compared with WT animals. In this way, the development of the retinas could be tracked as the disease progressed. This diachronic study covers a wider timeframe than any other study performed so far. Histological studies provide a wide spectrum of information, but the processing of retinal tissue for analysis causes thicknesses to vary, which makes it difficult to correlate directly with the data provided by current techniques like OCT, which allows us to analyze *in vivo* the changes experienced by the retina as the disease develops (Salobrar-García et al., 2016). In this study we demonstrated for the first time retinal changes from the early to late stages of AD using OCT in the APP^NL−F/NL−F^ model.

The APP^NL−F/NL−F^ model presents an age-related Aβ pathology, memory deterioration, behavioral problems, and neuroinflammation (Cash et al., 2013; Rochat et al., 2013; Saito et al., 2014; Masuda et al., 2016; Sasaguri et al., 2017; Shah et al., 2018) and allows us to estimate early changes in the retina.

It is now known that there are relationships between certain areas of the brain and the retina. Specifically, the thickness of peripapillary RNFL (pRNFL) in the temporal sector is related to the volume of the medial temporal lobes, especially with the volume of the hippocampus (Shi et al., 2020). In the lower sector the pRNFL thickness is associated with the volume of the occipital lobes and selectively with the volume of the lingual gyrus. Therefore, the cerebral changes observed in AD may be related to retinal changes and the retina may be useful as a biomarker of neurodegenerative diseases (Shi et al., 2020).

In APP^NL−F/NL−F^ model, at 3 months old, there was no presence of Aβ pathology, although there was already an alteration of the proteome in both the hippocampus and the cortex compared to WT mice. The early increase in Tris-soluble Aβ_42_ levels suggests that the pre-symptomatic stages of AD begin before amyloidosis Aβ (Schedin-Weiss et al., 2020). In addition, the accumulation of Aβ is age-dependent from 1 to 18 months (Petrache et al., 2019). Aβ brain deposits have been reported at 6 months of age in this AD model (Saito et al., 2014), developing first in the hippocampus and then becoming more significant in the cerebral cortex (Schedin-Weiss et al., 2020). At 9 months old, there was a significant amount of Aβ plaques in the parenchymal brain, with the Aβ plaque load reaching its maximum at 18 months (Schedin-Weiss et al., 2020). While the brain changes in this model have been studied, there are not works analyzing the retina. However, in other transgenic models retinal changes have been previously reported.

In the retina, the formation of Aβ plaques was described to occur at 6 months in the APP/PS1 model (Georgevsky et al., 2019), although other authors observed these plaques earlier in the retina at 2.5 months old (Koronyo-Hamaoui et al., 2011). Using the 3xTg-AD model, it was demonstrated that there is a positive correlation between retinal thickness and the volume of the visual cortex (Chiquita et al., 2019), as well as the behavior of the microglia in the retina, showing activation and migration between the different layers of the retina in whole-mount retinas (Salobrar-García et al., 2020). Histological alterations has been described in the retina, such as a decrease in the density of retinal ganglion cells and the nerve fiber layer, thinning of the inner plexiform layer, and the presence of Aβ plaques in the inner nuclear layer in a APP/PS1 model (Gupta et al., 2016).

Scarce studies have analyzed retinal changes using OCT in transgenic models (APP/PS1 and 3xTg-AD), however, to our knowledge, there is no study that analyses the retina in the APP^NL−F/NL−F^ model. By analyzing two horizontal lines above and below the optic nerve, Georgevsky et al. (2019) showed, in both APP/PS1 mice and WT controls, a significant age-related reduction in the inner retinal thickness from 3 to 12 months, with a significant difference between the APP/PS1 and WT mice in both the inner and outer retinal thickness starting at 9 months. In contrast, Harper et al. (2020) using multicontrast OCT, more recently found no significant changes in retinal thickness between APP/PS1 mice and control mice in any of the retinal regions analyzed by scanning an annulus around the ONH divided into two sectors. Finally, Song et al. (2020) using a multimodal imaging system with co-registered OCT and angle-resolved low-coherence interferometry, found a significant thinning of RNFL in 3xTg-AD mouse retinas compared to the WT controls.

Although the use of OCT gives us *in vivo* images at a very high resolution that allow us to analyze the retinal layers in animal model studies, most OCTs designed for animal studies have no tracker system. To date, no published studies have been conducted using this technique (Georgevsky et al., 2019; Harper et al., 2020). However, our diachronic study in APP^NL−F/NL−F^ model was conducted using SD-OCT with a tracking system that allowed us to re-examine the exact same area, thereby avoiding measurement errors or unintentional movements and giving us the ability to analyse areas of the retina that cover almost the entire posterior pole instead of a single scan line.

In APP^NL−F/NL−F^ mice the early retinal thinning observed with SD-OCT at 6 months of age (which was significant in total retinal thickness only in the outer temporal sector, as well as in the nasal, infero–temporal sectors and mean global value of the RNFL) appeared to develop toward more significant thinning by the final time point. It is possible that these initial changes are the consequence of the progressive accumulation of soluble oligomers of Aβ (in our model Tris-soluble Aβ_42_), inducing early neuronal dysfunction due to their toxicity. These changes are in line with the observation that the APOE ε4 genotype of AD is associated with a decrease in GABAergic interneurons and glutamatergic signaling in the hippocampus, which is a risk factor for AD (Andrews-Zwilling et al., 2010; Busche and Konnerth, 2016; Shah et al., 2018). In the APP/PS1 model, primary visual cortex degeneration has also been observed in parallel with an increase in Aβ plaque with age. This is specific to the hyperactive neurons located near plaques, which are also found in the frontal cortex in AD (Busche et al., 2008). The hyperactive astrocytes located in the vicinity of the Aβ plaques that are formed may also contribute to neuronal protection, which can directly improve neuronal activity initially. This astrogliosis becomes noticeable very early and correlates with the slow development of AD in the APP^NL−F/NL−F^ mice at 6 months (Saito et al., 2014; Petrache et al., 2019). The findings at this early time may also be secondary to the astrocyte reduction of glutamate synaptic recapture (Li et al., 2009) and to the excessive amount of Aβ dimers, as well as the Aβ_1−40_ monomers and dimers, which increase the presynaptic release of glutamate (Fogel et al., 2014). Therefore, a combination of both causes could increase residual glutamate levels, thereby promoting neuronal hyperactivity, which is a precursor to plaque formation (Busche and Konnerth, 2016; Schedin-Weiss et al., 2020).

In the APP^NL−F/NL−F^ mice, the time between 9 and 15 months of age was characterized by a slow and progressive tendency toward thickening of the retina with alternation of thinned retinal sectors, which vary over time and present only statistical significance at 15 months in some sectors of the total retinal thickness (outer and inner rings of inferior sector and inner ring of the temporal sector). These changes could correlate to the brain changes seen in this model at 9 months, when neuronal death was detected by necrosis in the cerebral cortex (Schedin-Weiss et al., 2020). At the same time, the effect of microgliosis and astrogliosis are significant in the APP^NL−F/NL−F^ model in the cortex, hippocampus, and subcortical region compared to the WT mice (Masuda et al., 2016). These mechanisms could explain why, at 9 months, although there were changes in the OCT analysis in the APP^NL−F/NL−F^ group compared to WT mice, these changes were not statistically significant since neuroinflammation could appears at the same time as neurodegeneration, masking the changes seen at 6 months of age (Saito et al., 2014; Masuda et al., 2016). These slow and minor changes in retinal thickness, which evolve steadily until 15 months of age, could be produced by the progressive deposit of Aβ plaques (Radde et al., 2006; Ferguson et al., 2013; Nilsson et al., 2014; Masuda et al., 2016; Stevanovic et al., 2017; Shah et al., 2018; Petrache et al., 2019; Schedin-Weiss et al., 2020) and by the mechanisms of inflammation, phagocytosis, and microglial migration between different retinal layers (Lee et al., 2020; Salobrar-García et al., 2020).

While in the APP/PS1 model, at 16 months of age, there was a heavy plaque burden throughout all cortical regions (Ferguson et al., 2013), as well as increased microglial activity (Perez et al., 2009), in the murine model used in our study, this heavy plaque load peaked at 18 months. At the same time, the mice showed alterations in different proteins of the hippocampus and the cortex, which are involved in various neuronal maintenance activities (Schedin-Weiss et al., 2020).

These findings may be linked to the overall increase in total retinal thickness at 17 months of age in the APP^NL−F/NL−F^ group, with significance in the nasal and inferior sectors, the inner and outer sectors, and the RNFL (albeit without statistical significance); these changes were more noticeable in the nasal–superior, nasal–inferior, and temporal–inferior sectors. At this point in our study, where the greatest thickening was observed, we precisely observed that Aβ brain plaques accumulate at an accelerated rate (Koronyo-Hamaoui et al., 2011), which coincides with the findings of our model (Saito et al., 2014; Schedin-Weiss et al., 2020).

In addition, astrocytes are hyperreactive, surrounding the Aβ plaques and releasing proinflammatory factors that induce higher microglial activation (Saito et al., 2014; Busche and Konnerth, 2016; Masuda et al., 2016; Shah et al., 2018). In the APP/PS1model there was an increase in the microglial marker F4/80, inflammatory cytokine MCP-1, and TUNNEL-positive cells in the RGC layer (Ning et al., 2008). In addition, the activated microglia may trigger a neuroinflammatory response (Perez et al., 2009) that could contribute to retinal disorganization, as demonstrated by the functional alterations present in the electroretinogram of the APP/PS1 mouse model (Krasodomska et al., 2010; Ramirez et al., 2017). In APP^NL−F/NL−F^ mice, high levels of Aβ_42_ cause pathological Aβ deposits in the cerebral cortex and hippocampus, which are accompanied by increased neuroinflammation, with activation of the astrocytes and microglia surrounding the plaques from 6 months of age (Saito et al., 2014; Sasaguri et al., 2017; Schedin-Weiss et al., 2020). In our study, Iba-1+ cells located in the OPL and IPL were larger, with thicker and larger somas and processes than in the WT group (Supplementary Figures 1D,F). In addition, in the GCL-NFL the Iba-1+ cells had a more amoeboid appearance with thicker somas and retracted processes (Supplementary Figures 1D,F). In the APP^NL−F/NL−F^ group there was an increase in GFAP+ immunostaining with astrocytes accumulating in areas where Iba-1+ cells also clustered (Supplementary Figures 1E,F). When the microglia is activated, it undergoes morphological changes and it is transformed into cells with an amoeboid appearance and phagocytic properties, capable of releasing substances that induce an inflammatory response (Rowland and Shneider, 2001). These morphological changes are gradual, ranging from the branched resting state to an intermediate, early activated or “primed” state, and finally reaching the amoeboid phagocytic state (Perry, 2004). In the “primed” state, the microglia increases its vigilance state and shows a thickening of the cell body and its processes. This change in the microglia can occur in response to primary factors derived from neurons or astrocytes. Astrogliosis has also been observed in patients with AD in the GCL (Grimaldi et al., 2018, 2019). All microglial and astroglial cell changes, which may be associated with the neuroinflammatory process, could result in the increased retinal thickness observed in APP^NL−F/NL−F^ group.

Finally, at 20 months of age, which is the latest time point we analyzed, the findings of SD-OCT show significant thinning of the total retina thickness in the inner ring of nasal, inferior and temporal sectors and in the outer rings of superior and temporal sectors, contrary to where the thickening occurred at 17 months, which could be the result of the generalized thinning of the retina. It should be noted that the outer temporal sector, the first sector with a significant change at 6 months, again showed significant thinning being more pronounced than that at earlier time points. The RNFL showed generalized thinning reaching statistical significance in nasal, supero-temporal sectors and mean global value. This situation could be a consequence of the neurodegeneration caused by high levels of Aβ and oligomers, which could be promoting neurotoxicity at this time point (Schedin-Weiss et al., 2020). At the same time, a decrease in retinal ganglion cell density due to apoptosis (Ning et al., 2008; Gupta et al., 2016), a decrease in the axon density of the optic nerve, and a significant thinning of the inner plexiform layer were found in the APP/PS1 model (Gupta et al., 2016).

Notably, in the SD-OCT analysis from 6 to 20 months of age, in the RNFL, the only sector that did not increase in thickness was the superior temporal sector, while that with the most fluctuations was the superio–nasal sector, which correlates with the temporal sectors in total retinal thickness because, again, the sector that exhibited the least thickness variation was the outer temporal sector. These structural changes are consistent with the functional findings of the electroretinogram (ERG), which show dysfunction of the RGCs and cones, as well as a response from bipolar and other interneuronal cells in the inner retina in the APP/PS1 mice (Gupta et al., 2016), which could support the diminished thickness of the retina detected in our APP^NL−F/NL−F^ mice.

It must be considered that this data should be corroborate in future works with molecular biological investigations that confirm the findings in this model.

In summary, the first changes observed at 6 months of age included a significant thinning of the total retinal thickness in the outer temporal sector, and significant thinning of the RNFL in the nasal and inferior–temporal sectors, and mean global value. At the later time points, which correspond to 9 and 12 months of age, there was a slow and progressive evolution toward thickening, with alternation of the thinned sectors, albeit without statistical significance. At 15 months of age, there was a significant thinning of the total retinal thickness in the inner rings of temporal and inferior sectors and in the outer ring of inferior sector. At 17 months of age there were a widespread thickening of the total retina, which was significant in the inferior and nasal sectors of both the inner and outer ring. This thickening could be due to a neuroinflammatory process produced by astrocytes and microglia changes. Finally, at 20 months of age, the SD-OCT showed generalized non-significant thinning of the RNFL and a considerable thinning of the total retinal thickness, with greatest significance in the superior and temporal sectors of both the inner and outer ring.

## Conclusions

In conclusion, this diachronic study of the murine model of APP^NL−F/NL−F^ of AD from 6 to 20 months of age provides significant information on the variations in total retinal thickness and RNFL measured by SD-OCT. In this model, the retinal thickness showed thinning, possibly produced by neurodegeneration alternating with thickening that could be caused by deposits and neuroinflammation in some areas of the retina.

Retinal changes over time, similar to those observed in the human retina could be a biomarker for AD. The APP^NL−F/NL−F^ AD model may help us better understand the different retinal changes during the progression of AD.

## Data Availability Statement

The datasets generated for this study can be found in online repositories. The names of the repository/repositories and accession number(s) can be found at: https://figshare.com/s/b3303423bc9cac47ac09.

## Ethics Statement

The animal study was reviewed and approved by Ethics Committee on Animal Welfare of the University Complutense (PROEX No. 047/16).

## Author Contributions

ES-G, IL-C, RdH, and JR: conceptualization. ES-G, IL-C, LS-P, JF-A, IB-F, VM, TCS, and TS: methodology. ES-G, IL-C, LS-P, and JF-A: investigation. ES-G, IL-C, and LS-P: formal analysis. AR and JS: project administration. AR, JS, MM, TCS, TS, RdH, and JR: funding acquisition. ES-G, IL-C, RdH, and JR: writing—original draft. ES-G, IL-C, AR, TS, JS, MM, RdH, and JR: writing—review and editing. All authors read and approved the final manuscript.

## Conflict of Interest

The authors declare that the research was conducted in the absence of any commercial or financial relationships that could be construed as a potential conflict of interest.
