# Supplementary material for: Retinal Thickness Changes Over Time in a Murine AD Model APPNL-F/NL-F
Source: Front Aging Neurosci. 2021 Jan 15;12:625642. doi: 10.3389/fnagi.2020.625642 (PMC7852550; doi:10.3389/fnagi.2020.625642)
Supplement: Supplementary file 1 [file Data_Sheet_1.docx]

**Supplementary data**

## Material and methods

## Immunohistochemistry

To analyse whether a neuroinflammatory process could be occurring in the retina, double immunofluorescence was made in 17-month-old animals from the two study groups: APP^NL-F/NL-F^ mice (n=3) and WT mice (n=3). We chose the 17-month-old animals because it is at this age at which thickening of the retinal tissue was first observed when studied with the OCT. Since neuroinflammation is orchestrated by the progressive activation of microglia and astrocytes in AD (Ahmad et al., 2019), we have used antibodies that specifically mark these cells, anti-Iba-1 for microglia labelling and anti-GFAP for astrocyte labelling.

To obtain the tissues, the mice were transcardially perfused through the ascending aorta with 0.1 M phosphate buffered saline (PBS) followed by 4% paraformaldehyde (PFA) in 0.1 M PBS. Perfusion was performed under general anesthesia administered by intraperitoneal injection of a mixture of xilacina (10 mg/kg; Rompún®, Bayer, Barcelona, Spain) and ketamine (75 mg/kg; Ketolar®, Parke-Davies, Barcelona, Spain). After the fixation, to maintain the orientation of the eye, a suture was made in the superior eyelid. In addition, the insertion of the rectus muscle and the nasal caruncle were also used as orientation markers. Once the eyeballs were removed they were postfixed in the same fixative (4% PFA in 0.1 M PBS) keeping them at 4ºC until the next day. The eyes were washed three times, 30 min each, in PBS. Afterwards, the corneas and lenses were extracted, by introducing the resulting optical cups, which contained the retinal tissue, into a cryoprotective solution containing 30% sucrose in PBS at 4 ºC for 24 h. Finally, the samples were embedded in a tissue freezing medium (Tissue-Tek® O.C.T.™ Compound, Sakura Finetek Spain, [Barcelona], Spain), always maintaining the anatomical references of the eyes to ensure the spatial orientation of the retinas. Following this, they were then frozen in isopentane cooled in liquid nitrogen, and kept at -80ºC until used.

For the immunohistochemical analysis, the optical cup with retinas was frozen sectioned with a Leica CM-3050 cryostat (Leica Biosystems, [Heidelberger], Germany) into 12 μm thick sagittal sections. The tissue sections were collected on gelatine coated slides, and air dried at room temperature for 60 minutes in order to increase the adherence of the sections to the slides. In the performance of the double immunofluorescence, the washings were done in PBS, pH 7.4, which contained 0.1% Triton X-100 (washing buffer (WB)), and the incubations were done in PBS, pH 7.4, 0.1% Triton X-100 and 10% R.T.U. animal-free blocker and diluent, (SP-5035; Vector Laboratories, Inc., [CA], USA) (immunohistochemical buffer (IB)). After three washings in WB, the sections were incubated overnight at 4°C with the primary antibodies: rabbit anti-Iba-1 (ref. 019-19741Wako, Osaka, Japan) at 1:700 dilution and chicken anti-GFAP (ref. AB5541 Merck KGaA, [Darmstadt], Germany) at 1:50 dilution. Then, the sections were washed three times in WB and incubated for 3h at room temperature with the secondary antibodies: donkey anti-rabbit antibody conjugated to Alexa Fluor 594 (ref. A21207 Invitrogen, Paisley, UK) diluted 1:900 in PBS and donkey anti-chicken antibody conjugated to Alexa Fluor 488 (ref. 703-545-155 Jackson Immunoresearch, Cambridge UK) diluted 1:500 in PBS. The nucleus of different retinal cells was stained with DAPI, in a 1:1000 dilution; this helped us to be better orientated throughout retinal thickness. After incubations, sections were then washed three more times with WB, and finally coverslipped with a Vectashield Vibrance Antifade® mounting medium with DAPI (Ref. H-1800; Vector Laboratories, [CA], USA). Three negative controls were performed to check that the secondary antibodies react with their corresponding primary antibodies. In the first control, the primary antibody was not added, and the tissue was incubated with the primary antibody diluent and then with the secondary antibody. In the second control, the secondary antibody was omitted and incubated only with the primary antibody and the secondary antibody diluent. In the third control, neither the primary nor the secondary antibody was placed, and the tissues were incubated in the corresponding diluent solutions to evaluate the amount of endogenous fluorescence in the tissue.

Immunostained slides were observed under a fluorescence microscope Zeiss Axio Imager M.2 (Carl Zeiss AG, Oberkochen, Germany) associated with the Apotome-2 module (Carl Zeiss AG, Oberkochen, Germany) and high-resolution camera Axio Cam 503 Mono (Carl Zeiss AG, Oberkochen, Germany). The microscope was equipped with a Zeiss 10 filter set for Alexa Fluor 488, a Zeiss 64 filter set for Alexa Fluor 594, and a 49 filter set for Alexa Fluor 405. The images taken were analyzed using ZEN2 software (Carl Zeiss AG, Oberkochen, Germany). All lighting conditions and magnifications were kept constant during the capture process. Figures were prepared using Adobe Photoshop CS4 Extended 10.0 (Adobe Systems, [CA], USA).

## Results

## Microglial and astroglial inmmunostaining in Wild Type and APP^NL-F/NL-F^ groups at 17 months old

Microglial and astroglial activation was evaluated in the WT and APP^NL-F/NL-F^ groups at 17 months old, by analysing the vertical retinal sections immunostained with Iba-1; a constitutively expressed microglia specific marker, and GFAP; a specific marker of the astrocytes that is the constituent of their intermediate filaments.

In the WT group, the Iba-1+ cells had the typical “resting” microglia appearance, with small cell bodies and delicate processes. These cells were located in the OPL, IPL and GCL-NFL (Supplementary Figure A, C). In addition, the GFAP+ immunolabelling, typical of astrocytes, was observed as a thin, discontinuous line in the NFL (Supplementary Figure B, C).

In the APP^NL-F/NL-F^ group of 17-month-old animals, the Iba-1+ cells located in the OPL and IPL were larger, with thicker and larger somas and processes than in the WT group (Supplementary Figure D, F). In the GCL-NFL the Iba-1+ cells had a more amoeboid appearance with thicker somas and retracted processes (Supplementary Figure D, F). These cells were grouped at different points in the GCL-NFL (Supplementary Figure D,F). The GFAP+ labelling also showed changes in the APP^NL-F/NL-F^ group. An increase in GFAP+ immunostaining was observed, with astrocytes accumulating in areas where Iba-1+ cells also clustered (Supplementary Figure E, F).

**Supplementary figure legend**

Immunohistochemical study of Iba-1 and GFAP expression in WT and APP^NL-F/NL-F^ mice at 17 months of age. Retinal sections were immunolabeled with antibodies to Iba-1 (Red), GFAP (Green), DAPI (blue) and merged (yellow).

**(A-C): WT group. A, C:** Iba-1+ cells had the typical “resting” microglia appearance, with small cell bodies and delicate processes. These cells were located in the OPL, IPL and GCL-NFL**. B, C:** GFAP+ immunolabelling, typical of astrocytes, was observed as a thin, discontinuous line in the NFL.

**(D-F): APP^NL-F/NL-F^ group. D, F:** Iba-1+ cells in the OPL and IPL were larger, with thicker and larger somas and processes than in the WT group (A). In the GCL-NFL, Iba-1+ cells had a more amoeboid appearance with thicker somas and retracted processes, grouped at different points in the GCL-NFL. **E, F:** In the APP^NL-F/NL-F^ group an increase in GFAP+ immunostaining was observed, with astrocytes accumulating in areas where Iba-1+ cells also clustered.

**Abbreviations:** Iba-1 (Ionized calcium-binding adaptor molecule 1), GFAP (glial fibrillary acidic protein); DAPI (4′,6-diamidino-2-phenylindole); WT (wild type); ONL (outer nuclear layer); OPL (outer plexiform layer); INL (inner nuclear layer); IPL (inner plexiform layer); GCL (ganglion cell layer); NFL (nerve fiber layer).
