# Supplementary figures and images for: Retinal Thickness Changes Over Time in a Murine AD Model APPNL-F/NL-F
Source: Front Aging Neurosci. 2021 Jan 15;12:625642. doi: 10.3389/fnagi.2020.625642 (PMC7852550; doi:10.3389/fnagi.2020.625642)

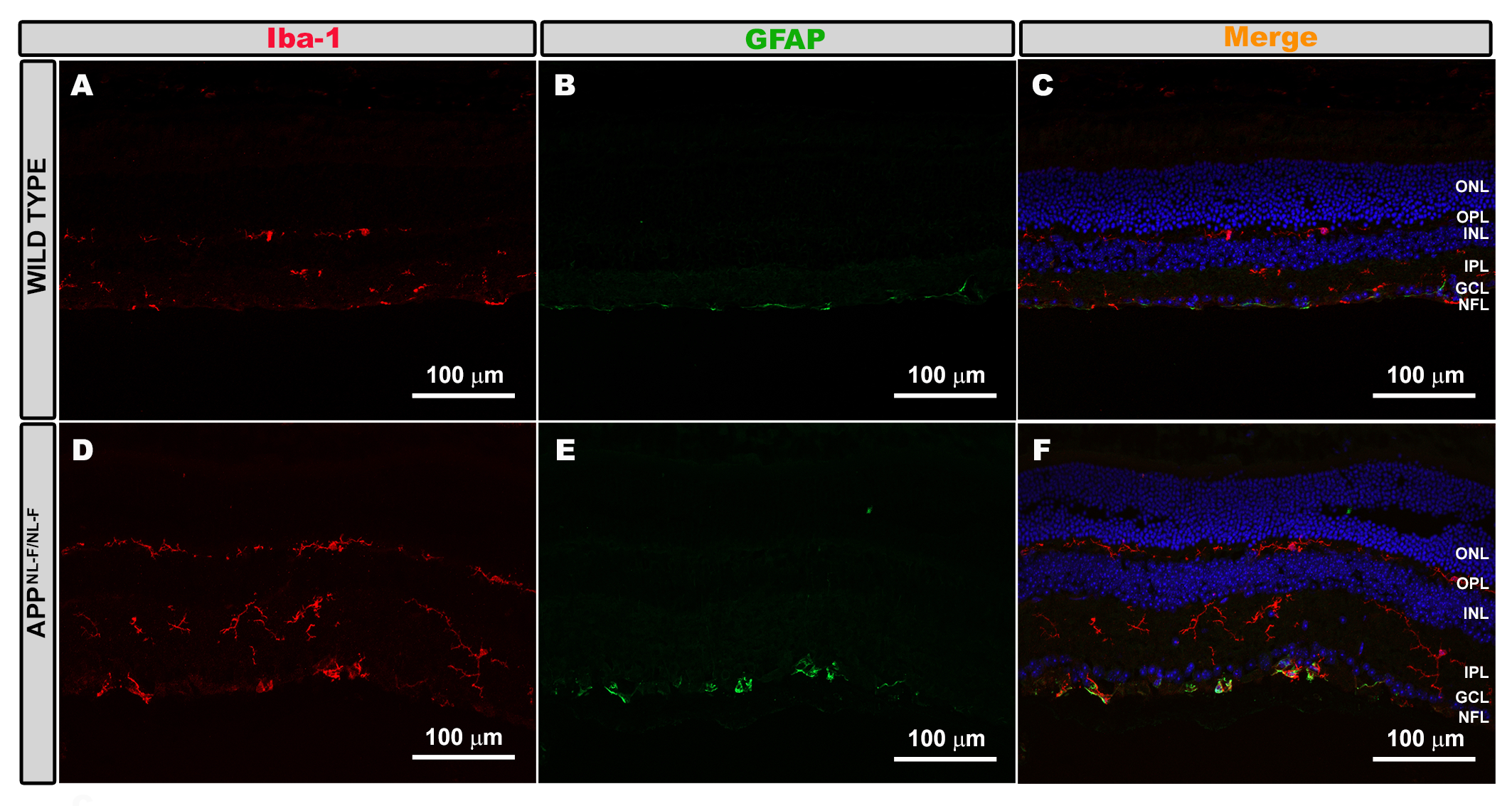

Supplement: Supplementary file 2 [file Image_1.TIF]
